# The Problem of Poor-Law Infirmaries: The Workhouse Influence

**Published:** 1917-03-24

**Authors:** 


### THE MATRONS' AND SISTERS' DEPARTMENT.

*[The claim by the National Poor-Law Officers' Association to represent trained nurses and that the nurses under the Poor Law exceed in numbers all other nurses, so entitling them to special representation on the College Council, renders it desirable to make the true position realised by publishing the facts. Poor-Law people have a habit of calling all sorts of persons nurses who are not trained nurses and are not eligible to apply for registration with the College. These claims demonstrate, too, the misapprehensions under which the Poor-Law Officers' Association and those connected with it appear to labour in this connection. An examination into the facts has produced the following article on the Workhouse Influence, which we publish as received. It should settle the claim of the Poor-Law Officers' Association to represent trained nurses, by its rejection, and induce every fully-trained Poor-Law nurse to decide to register with the College and not to join the Poor-Law Officers' Association.]*

### THE PROBLEM OF POOR-LAW INFIRMARIES.

### The Workhouse Influence.

Well-meaning persons who do not know the circumstances are often apt to ask why a point should be made of separating infirmaries from the workhouse, and to consider the question to be mainly one of the nurse's social position. No doubt this is one of the objections, but the real difficulty lies much deeper, and the influence and authority of the workhouse is fatal to really good training, unless it is continually kept at bay.

First as regards the patients. Nurses, under the workhouse influence, tend to become rough, contemptuous, and rude in manner to their patients, exactly as the average workhouse officer is to the "inmates" under his charge. The inmate himself, or herself, briefly describes it as "speaking to us like dogs." Nurses also become careless about nice and refined ways with their patients, and are lowered to their standard, instead of raising them to a higher one. Also, without exactly neglecting the patients, the workhouse point of view is certainly that their comfort is subordinate to that of the nurses. As regards the children, the workhouse influence leads the nurse to rough ways, to carelessness about proper manners and nice behaviour in the children, and indifference about their happiness, though not, as a rule, to wilful unkindness. A young child, coming to a well-managed children's ward from an average workhouse school, has often to be taught to smile and speak, as he only nods his head when spoken to, and has never been played with, or had his intelligence developed. Many of these children are quite wrongly thought to be mentally deficient. With regard to infants they are only properly nursed and attended to in really good infirmary wards. In the average workhouse they are regarded with complete indifference.

2. With regard to the nurse herself, the workhouse influence does all in its power to destroy her professional ideals. The nurse is a workhouse officer, the nurse of the So-and-so Union, and any attempt on the superintendent-nurse's part to encourage professional feeling among her nurses is promptly opposed and, if possible, suppressed by the Guardians and workhouse officials. Yet the consciousness that she belongs to a great profession, and must live up to its highest ideals, is the greatest secondary motive that a nurse can have for thoroughness and devotion in her work.

3. Divided authority is another very unfortunate result of the workhouse connection. The nurses have an appeal from the superintendent-nurse to the workhouse master, and this power is used exclusively by the unsatisfactory nurse. Steady, sensible nurses do not take their difficulties to the master. But the power of appeal makes discipline exceedingly difficult, as the master is always inclined to support the insubordinate nurse. The constant interference of the workhouse matron is even more vexatious, as the pain of a wound is often easier to bear than the constant pricking of a pin. The matron's authority is displayed in small ways. She has charge of all stores and linen, and often differs from the superintendent-nurse as to the amount of clean linen required for the wards. She can also make constant difficulties about all domestic arrangements for the nurses, and the cleaning of the wards. This all sounds very trivial, but a constant system of interference about trifles causes much greater wretchedness than one or two serious troubles, which can be settled, once for all, in a short time.

4. The professional and social position of infirmary nurses is not so good as that of women, of exactly the same class, trained in general hospitals. The result of this is that the better class of candidate hesitates to apply, even to large infirmaries. Excellent people consider that any good rough girl will do very well for a "workhouse nurse." Many people think that infirmary nurses are simply attendants on old people, and say with surprise to the matron of a large training-school, "But I

suppose you have no *sick*, have you?" At present, even first-rate infirmary nurses have to justify their claim to be regarded as equal to nurses trained in general hospitals.

5. The attitude of the Guardians towards the infirmary makes training and discipline much harder. In many infirmaries the probationers are chosen by the Guardians, and often are personally known to individual members of the Board. This is bad for the probationers, as the Guardian "looks after" the nurse he knows—that is, tries to get privileges for her that other nurses cannot have; and in return expects to hear all the gossip of the infirmary. If the superintendent resists this, and is strictly just to all her nurses, the Guardian can make himself very unpleasant by opposing the superintendent in any request she makes to the Board. If she yields, she loses the confidence and respect of her nurses, and makes jealousy among them. The Guardians have a tendency to ignore the training-school, and to regard the infirmary as only an inferior part of the workhouse. In the case of large unseparated infirmaries especially, the Guardians may not have the least idea that the "sick-wards" they think so little of are really a training-school, well known and highly thought of all over the professional world.

Half-measures are only palliatives against these difficulties. Partial separation—that is, keeping the workhouse matron out of the infirmary; entire separation—that is, putting the infirmary associated with the workhouse under a medical superintendent; these measures are really only treating symptoms. The disease is the connection of the Poor-Law infirmary, in any form, with the Guardians and the workhouse. Nothing but a complete separation, and the turning of the Poor-Law infirmary into a State hospital, would really meet the case. The State hospital nurse would then take her own honourable place, as a member on an equality with all other members of the great profession of nursing.

